# Quartz Enhanced Photoacoustic Spectroscopy on Solid Samples

**DOI:** 10.3390/s24134085

**Published:** 2024-06-24

**Authors:** Judith Falkhofen, Marc-Simon Bahr, Bernd Baumann, Marcus Wolff

**Affiliations:** 1 Heinrich Blasius Institute of Physical Technologies, Hamburg University of Applied Sciences, 20999 Hamburg, Germany; marc-simon.bahr@haw-hamburg.de (M.-S.B.); bernd.baumann@haw-hamburg.de (B.B.); marcus.wolff@haw-hamburg.de (M.W.); 2 School of Computing, Engineering and Physical Sciences, University of the West of Scotland, Scotland High Street, Paisley PA1 2BE, UK

**Keywords:** photoacoustic spectroscopy, IR, QEPAS, solid samples, higher harmonics, resonator design, FE-simulation, MEMS microphone, ultrasound

## Abstract

Quartz-Enhanced Photoacoustic Spectroscopy (QEPAS) is a technique in which the sound wave is detected by a quartz tuning fork (QTF). It enables particularly high specificity with respect to the excitation frequency and is well known for an extraordinarily sensitive analysis of gaseous samples. We have developed the first photoacoustic (PA) cell for QEPAS on solid samples. Periodic heating of the sample is excited by modulated light from an interband cascade laser (ICL) in the infrared region. The cell represents a half-open cylinder that exhibits an acoustical resonance frequency equal to that of the QTF and, therefore, additionally amplifies the PA signal. The antinode of the sound pressure of the first longitudinal overtone can be accessed by the sound detector. A 3D finite element (FE) simulation confirms the optimal dimensions of the new cylindrical cell with the given QTF resonance frequency. An experimental verification is performed with an ultrasound micro-electromechanical system (MEMS) microphone. The presented frequency-dependent QEPAS measurement exhibits a low noise signal with a high-quality factor. The QEPAS-based investigation of three different solid synthetics resulted in a linearly dependent signal with respect to the absorption.

## 1. Introduction

Photoacoustic spectroscopy (PAS) is based on the absorption of modulated laser radiation and the generation of a sound wave. In gaseous samples, light energy is absorbed by transforming it into molecular vibrations, rotations or electronic excitations, depending on the wavelength of the light (i.e., its energy). The modulated excitation causes local pressure changes that generate a sound wave with the same frequency as the light modulation.

A variety of acoustic detectors are available to record photoacoustic signals. High-precision measuring microphones, MEMS microphones or ultrasonic detectors, and piezoelectric sensors are used in photoacoustic sensors. Piezoelectric sensors can be made of different materials like polyvinylidene fluoride, lead zirconate titanate or polypropylene films and quartz crystals [1]. Quartz tuning forks (QTFs) are micromechanical tuning forks made of silicon dioxide. They vibrate at their maximum when exited with their resonance frequency, generating a voltage corresponding to the charge displacement caused by the vibration. Standard tuning forks for quartz watches exhibit a resonance frequency of 32,768 Hz. QTFs have the following advantages [1,2,3,4]:

- They separate noise from the rest of the signal and, therefore, can reach a higher specific sensitivity than microphones.
- They are temperature-stable over a wide frequency range and insensitive to magnetic fields.
- They are inexpensive and miniaturised, making them suitable for mass production.
- They are more robust than other piezoelectric sensors.
- They typically have high resonance quality factors.

Quartz-Enhanced PAS (QEPAS) combines the advantages of QTFs with those of photoacoustic spectroscopy. So far, this technique has been exclusively used for gaseous samples. A variety of configurations have been thoroughly tested, with the most common being the on-beam configuration. This involves the light passing through the gap between the prongs of the QTF. To amplify the signal, the QTF is often positioned between two identical cylindrical resonance tubes.

In the off-beam configuration, the sound pressure wave is detected at a certain distance from the laser beam. The QTF is here typically positioned outside a resonance tube at a narrow opening. This slit or hole has a width on the order of the spacing of the prongs. There are other off-beam approaches with two tubes arranged in a T-shape [5]. Implementations using two parallel tubes with facing slits and a single QTF [6] aim for increased amplification. Alternatively, two T-shaped resonators with opposite openings were tested with the QTF in between. Since each prong was facing an opening, the sound hit the QTF from both sides [7]. Other tested resonator geometries include H-shaped cells [8], Helmholtz resonators [9], elliptical tubes [10] or semicylindrical resonators [11].

Apart from theoretical considerations of a tuning fork immersed in a liquid medium [12] and using a QTF to optically analyse solid samples with so-called light-induced thermoelastic spectroscopy (LITES) [13], there are currently no studies of QEPAS on solid or liquid samples. To our knowledge, we performed the first QEPAS measurements on a solid sample and designed a special cell for this purpose. The requirements for such a cell differ from those for gaseous samples.

The following section briefly describes the photoacoustic effect of solids. After that, we present the new cell design, which is supported by a three-dimensional Finite Element simulation. Section 4 shows the experimental methods and results, including the characterisation of the cell resonance using an ultrasound (US) microphone and the QEPAS measurements. A discussion and an outlook are presented in the concluding Section 5.

## 2. Photoacoustic Effect of Solids

In solid samples, the absorption of periodically modulated optical energy leads to lattice vibrations or phonons. The resulting heat modulation has the same frequency as the light modulation and propagates to the sample boundary. A thin layer of the coupling medium in front of the sample acts like a piston on the rest of the volume, causing periodic pressure fluctuations in the gas. Thus, a photoacoustic (PA) signal is generated by a periodic temperature flow from the sample surface to an adjacent gaseous medium, which should exhibit low absorption in the spectral excitation range [14,15]. In contrast to traditional absorption spectroscopy, PAS is not affected by scattered light, which can cause inaccurate results when investigating solid samples. Additionally, PAS can also be used to analyse completely non-transparent samples [16].

In the case of an optically opaque and thermally thick sample, the acoustic sound pressure fluctuation δP as a result of the movement of the gas piston depends on the time t and the circular modulation frequency ω [14].

$$\delta P(t)=Qe^{j(\omega t-\pi /4)}$$

The factor Q is as a function of the thermal diffusivity of the gas in front of the sample $\alpha_{g}$ (air) and the following properties of the sample:

- optical absorption coefficient β,
- thermal diffusivity of material $\alpha_{s}$,
- thermal diffusion length in the sample (${\;\mu }_{s}$ = $\frac{1}{\sqrt{\omega /2\alpha_{s}}}$),
- thermal conductivity of the material $k_{s}$ [17].
  $$Q=-\left(j\frac{\beta \mu_{S}}{2(\sqrt{\omega /2\alpha_{g}})}\right)\left(\frac{\mu_{s}}{k_{s}}\right)Y$$

The factor Y depends on the incident light power $I_{0}$, the length of the gas piston $l_{g}$ being equal to the length of the cylindric resonator adjacent to the sample, the ratio of the specific heats in the adiabatic gas law γ and the overall temperature $T_{0}$ (ambient temperature and the DC-component at the solid surface due to temperature rise) and the ambient pressure $P_{0}$ [17].

$$Y=\frac{\gamma P_{0}I_{0}}{2\sqrt{2}l_{g}T_{0}}\;$$

This shows the photoacoustic signal is dependent on the absorption and thermal properties of the sample as well as on ambient parameters such as air temperature and thermal air diffusivity. The signal increases with increasing absorption in the sample and increasing light intensity.

The depth of penetration depends on the absorption coefficient of the sample and the incident light energy, which decreases proportional to $\beta^{-1}$. The thermal energy generated by absorption contributes only up to the simple thermal diffusion length $\leq \mu_{s}$ to the photoacoustic signal [14].

## 3. QEPAS Cell for Solid Samples

A semi-open cylindrical cell for the QEPAS analysis of solid samples has been developed. The manufacturing process for the cell is not only simpler compared to cuboid or T-/H-shaped cells [18,19,20,21]; the PA signal generation is also more efficient because the aforementioned geometries exhibit pipe diameter changes, which results in additional damping due to impedance discontinuities, leading to significant energy losses [22].

### 3.1. Cell Design

The new cylindrical cell is tightly sealed at one end by the solid sample and thus represents a half-open cylinder. The open end enables free access to the sample for the laser beam. Closed ends result in a 180-degree phase shift of the reflected sound wave, while open ends do not cause a phase shift. The resonance frequencies $f_{m}$ of the half-open cylinders are described by the equation [23].

(1) $$f_{m}=\frac{\left(2m-1\right)c}{4\;(l+∆l)}\;\mathrm{with}\;m=1,\;2,\;3\ldots$$

where c is the sound velocity, l is the geometric length of the cylinder and ∆l is the end correction which mainly depends on the radius of the cylinder a and can be calculated from [24].

(2) ∆l=0.6133·a.

**Figure 1:**
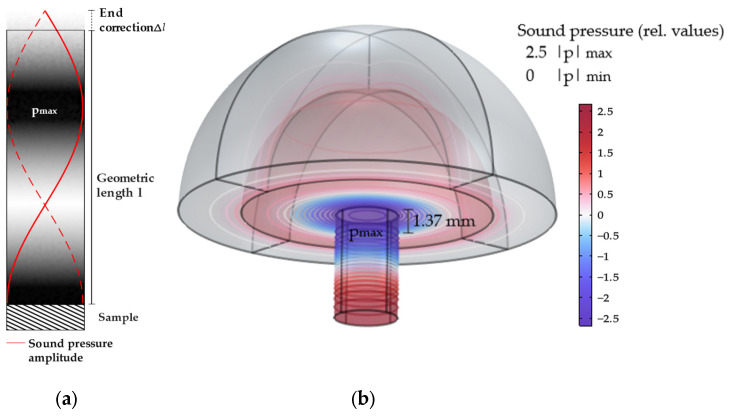
(**a**) Sound pressure distribution of the second harmonic longitudinal resonance in the centre plane of a half-open cylinder. (**b**) 3D sound pressure distribution of the second harmonic longitudinal resonance of a half-open cylinder. Dark blue and dark red corresponds to the pressure anti-nodes (180 degrees out of phase).

For a maximum photoacoustic signal, modulating the radiation with a frequency that corresponds to the most intense acoustic resonance is recommended. Here, we use the first overtone, which is also called the second harmonic (m=2), as the fundamental leads to a maximum directly at the sample and higher modes have an increasingly lower intensity [25]. Figure 1a shows the corresponding sound pressure distribution.

In the experiments described in Section 4, a commercial QTF will serve as a sound detector for the PA signal. Its nominal resonance frequency is $f_{R}=32.768$ kHz. Since the sample is excited by a laser beam, the diameter of the cylinder must be larger than the beam width. The laser beam should not hit the wall of the tube. Otherwise, a wall signal will be generated that will interfere with the desired sample signal. In order to fulfil this requirement, a tube radius of a=2.000 mm was selected, which is significantly larger than the laser spot size of ca. 0.1 mm.

Using Equations (1) and (2) and taking into account the second harmonic $f_{2}$, which corresponds to the QTF resonance frequency $f_{R}$, a geometric length of l=6.624 mm is obtained. The value 343 ms was used for the speed of sound.

### 3.2. Finite Element Simulation

We calculated the length of a cylindrical cell that can serve as a half-open resonator for solid samples. Previously, a 2D model was developed employing COMSOL Multiphysics^®^ to determine the length of the cylinder and the location of the opening [20]. Subsequently, we performed a 3D finite element (FE) simulation. The homogeneous Helmholtz equation was solved according to the acoustic sound pressure field. One condition was that the sound pressure at the walls must be maximum, and therefore, its normal derivative must be zero. It was assumed that the sample represented a soundproof seal of the tube. The particular difficulty of the open end was solved with perfectly matched layers (PML). Using an iterative approach, we determined the length of the cylinder at which the first overtone matched the resonance frequency of the QTF.

The additional dimension required more computational effort but is considered to be more accurate. Figure 1b shows the 3D sound pressure distribution of the second harmonic longitudinal resonance of the half-open cylinder.

The hemisphere attached to the top of the resonator represents the external space that integrates the PML. The sound pressure of the acoustic mode spreads into the external space, which makes the end correction necessary for the analytical calculation. The sound pressure distribution shows that the second harmonic results in two maximum amplitudes. One is located 1.37 mm from the open end and is determined as the location of the signal detection.

The 3D simulation confirms the previous 2D results [23]. There was no significant difference in the sound pressure distribution of the first overtone. However, the accuracy has improved due to fewer approximations and the consideration of azimuthal and radial modes at higher frequencies.

Table 1 shows the results of the analyses regarding the dimensions of the cylinder geometry. Compared to the analytical calculation, the required cell is, according to the FE simulation, 0.55% (0.037 mm) larger.

## 4. Experimental Investigations

### 4.1. Experimental Setup

The cylindrical PA cell was made of stainless steel with a wall thickness of 0.100 mm. The accuracy of the cell length equals 0.005 mm. The optimal location for the sound detector is where the maximum sound pressure occurs. In order to achieve this as best as possible with minimum distortion of the sound field, a hole of 0.250 mm diameter is laser-drilled into the cell wall, 1.370 mm from the open end. The sound detector is then placed outside the cell, closest to the hole.

For the verification of the cell resonance presented in Section 4.2, an ultrasound (US) micro-electromechanical system (MEMS) microphone is utilised [26]. For the QEPAS measurements presented in Section 4.3, the microphone is replaced by a QTF. Custom-made electronics based on high-precision differential amplifiers boost the respective signals.

In both investigations, a graphite disk serves as a test sample. This carbon material represents a spectrally broadband absorber. At the wavelength of 3380 nm, the material exhibits a relatively high absorption value for a solid, which is advantageous for the initial functionality test of our sensor. The absorption of the material for the given frequency was measured using FTIR-ATR (ThermoFisher Scientific Nicolet iS5, Waltham, MA, USA), resulting in an absorbance of 0.265, equivalent to 54.33% transmission. Its surface was repeatedly sanded with fine grit to achieve a soundproof seal with the cell.

**Figure 2:**
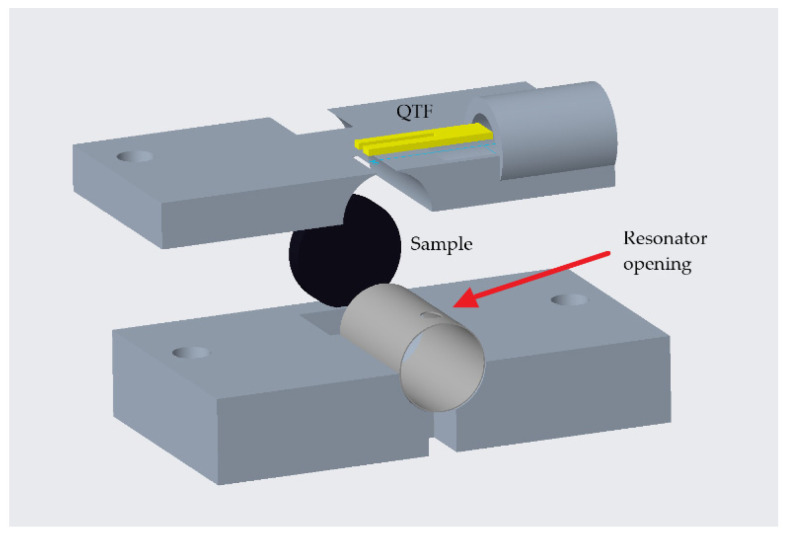
The mounting frame (in the exploded view) for the PA cell and the sample in the version for the QTF (the laser-drilled hole is marked with an arrow).

A mounting frame for the resonance cell and the sample was designed and 3D-printed from clear resin material. The version for the QTF is schematically depicted in Figure 2. The laser-drilled hole is marked with an arrow.

**Figure 3:**
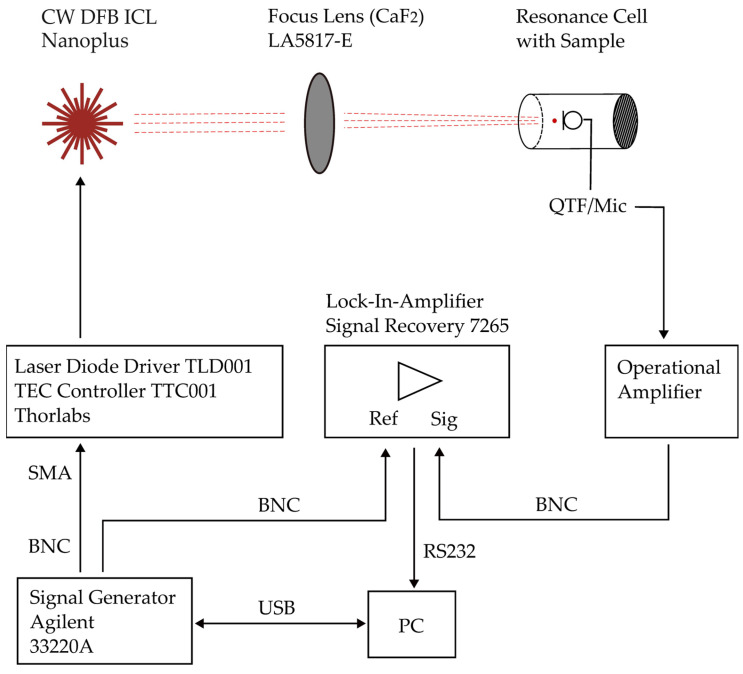
Experimental setup.

Figure 3 shows the experimental setup. A continuous wave (cw) distribute feedback (DFB) interband cascade laser (ICL) is used to excite the sample. Its emission is focused onto the sample surface.

The laser is regulated to a constant temperature of 20 °C by a thermoelectric cooler (TEC). A laser diode driver, in combination with a signal generator, modulates the laser between threshold and maximum current/maximum optical output power. A customised Matlab script automatically tunes the modulation frequency. A lock-in amplifier detects the PA signal. The complete setup is controlled by a PC that records the PA signal as a function of frequency. Table 2 provides specifications of the experimental setup.

### 4.2. Cell Resonance Verification

**Figure 4:**
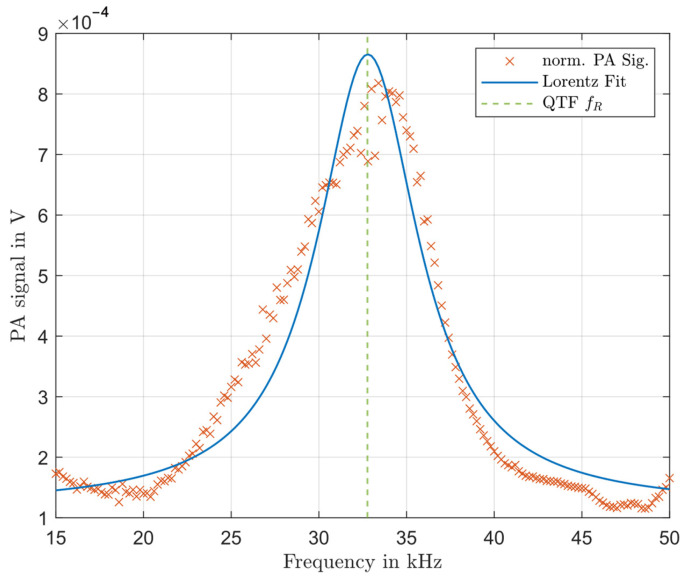
Normalised microphone signal of the resonance cell as a function of the modulation frequency of the laser together with a fitted Lorentz profile and the QTF resonance frequency.

Prior to the QEPAS measurements, the resonance frequency of the new PA cell, which was determined by FE simulation (Section 2), should be verified. For this, we measured the PA signal generated by the sample with the US MEMS microphone as a function of the modulation frequency of the laser. The cell was filled with air at 22 °C and 48% relative humidity and ambient air pressure of 101.325 kPa. None of the air components show significant absorption in the laser’s spectral range. Subsequently, we normalised the measurement according to the microphone’s transfer function [26]. Figure 4 displays the results. The normalised PA signal is represented by crosses. A Lorentz profile was fitted to the result, and the QTF resonance frequency is indicated by the dashed vertical line. Due to the challenges of the open resonator, a relatively low-quality factor of q = 4 is still achieved (3 dB-bandwidth of 4 kHz).

The Lorentz profile’s maximum corresponds almost precisely with the QTF resonance. The slight shift of only 0.1% to higher frequencies is likely due to the increased speed of sound at higher temperatures. The measurement, therefore, strongly supports the simulation results, demonstrating the accuracy of our methods.

### 4.3. QEPAS on Solid Samples

**Figure 5:**
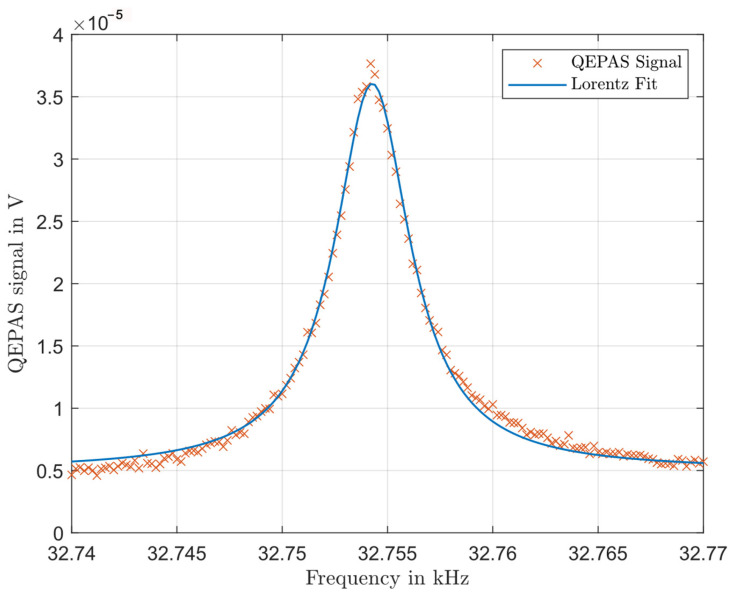
QEPAS signal of a solid sample as a function of the modulation frequency of the laser.

A standard QTF with a resonance frequency of 32,768 Hz was used for the QEPAS investigation. Its electronic original q-factor listed by the manufacturer (cf. Table 2) is 60,000 in a vacuum. This is lowered to around 10,000 due to the damping of air [13]. The precise positioning of the QTF is particularly important in this special off-beam configuration. The sound pressure wave should hit the upper third of the prongs [27]. Furthermore, both prongs should have the exact same distance to the sound source. Otherwise, one prong is more deflected than the other, which leads to asymmetries in the resonance function [28]. Additionally, the QTF should be positioned close to the hole in the cylinder to minimise the loss of sound pressure energy into the external space. However, if the QTF is placed too close, the signal could be reduced due to squeeze damping [29]. In order to achieve the best possible positioning, the QTF was mounted to a three-axis displacement unit and then placed approximately 0.5 mm from the laser-drilled hole parallel to the resonator wall. The centre of the hole was 2.6 mm below the QTF tip. To prevent direct excitation of the QTF by the laser (light-induced thermoelastic spectroscopy—LITES), it was shielded with an iris (Thorlabs ID15/M). Figure 5 displays the QEPAS signal of the solid sample as a function of the modulation frequency of the laser. The resonance curve is consistently smooth and exhibits only low noise. Figure 5 also shows a Lorentz fit, which is in good agreement with the measured data. It results in a resonance frequency of 32,754.2 Hz and a resonance width of 4.72 Hz (FWHM). This corresponds to a quality factor of q= 6.939. The QTF’s nominal resonance frequency is 32,768 Hz, with the manufacturer stating deviations of up to 5 ppm.

**Figure 6:**
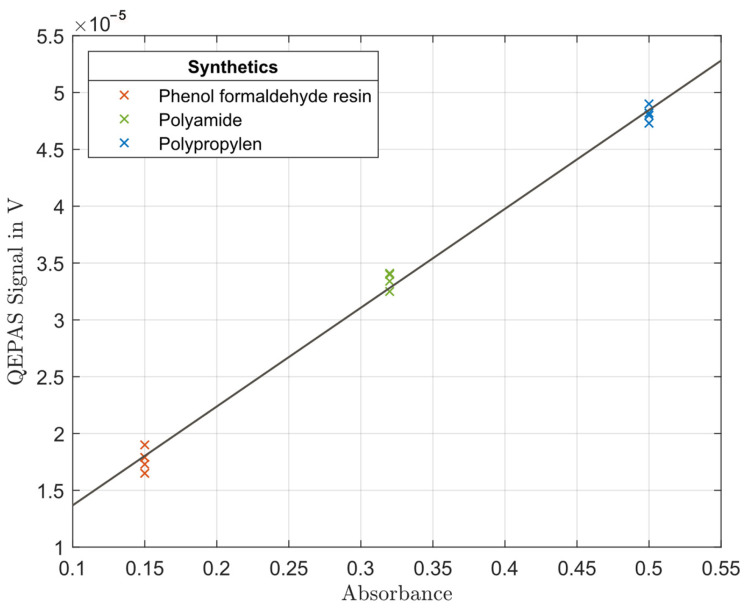
QEPAS signal for three different solid synthetics as a function of absorbance.

To investigate the dependency of the sensor signal on the sample absorbance, we measured the QEPAS signal of the following three different solid synthetics: phenol formaldehyde resin, polyamide and polypropylene. They cover a relatively wide range of absorbance [30]. Figure 6 shows the results together with a linear fit. The signal increases continuously with increasing absorbance.

## 5. Conclusions

We presented the first QEPAS measurement on a solid sample. A cw DFB ICL with a wavelength of around 3380 nm served as a light source. A disk made of graphite represented the sample.

A special cell has been developed based on the second harmonic of the fundamental longitudinal resonance of a half-open cylinder. The cell exhibits an acoustical resonance at the resonance frequency of the QTF in order to additionally amplify the PA signal and, thus, enhance the detection sensitivity. The required cell length was determined analytically and verified numerically using an FE simulation. A measurement with an ultrasound MEMS microphone confirmed the resonance frequency of the cell.

A tiny hole in the cell wall at the location of the pressure anti-node allowed the sound detector to access the sound pressure maximum outside the cell with only minimal distortion of the sound field. By tuning the modulation frequency of the laser, we were able to measure the ultrasound resonance function of the QTF around 32,754 Hz. The resonance quality was approximately 7000. A subsequent investigation of the QEPAS signals of three different solid synthetics shows a linear relationship between absorbance and signal amplitude.

The results open up completely new areas of application for the powerful QEPAS technology, as the new sensor can be adapted to a variety of scientific or industrial applications that require the investigation of solid samples.

However, there are still some challenges that simultaneously represent opportunities for future research. Precise sensor positioning under different environmental conditions is, for instance, crucial to achieving optimal performance and application-specific calibration. In order to adapt the QTF to a given cell, it could also be advantageous to use a custom-made tuning fork [31,32,33].

## Figures and Tables

**Table 1:** The geometry of the half-open cylindrical cell.

Cell Length		$\mathrm{Location}\;\mathrm{of}\;p_{\max}$
Analytical	FE	
l=6.624 mm	$l_{FE}=6.661\;\mathrm{mm}$	1.370 mm

**Table 2:** Specifications of the experimental setup.

Device	Description	Technical Detail	Value
Cw DFB ICL	Nanoplus S/N 1638/1-19	Operating temperature	20 °C
		Operating current	$I_{\mathrm{avg}}+I_{\mathrm{mod}}\cdot \sin\left(\omega t\right)=\;$ $52.5\;\mathrm{mA}+17.5\;\mathrm{mA}\cdot \sin\left(\omega t\right)$
		Typ. Wavelength	3380 nm
		Typ. Output power	8.8 mW
ICL Diode driver	Thorlabs TLD0011	LD Current noise	Typ. < 3 µA rms
TEC Controller	Thorlabs TTC001	-	-
Function generator	Agilent 3320A	Sine waveform	32,740…32,770 Hz
US MEMS Microphone	Knowles Analog bottom port: SPW0878LR5H-1 Ellen	SNR	65 dB(A) @ 1 kHz (differential mode) 76 dB @ 19 kHz (BW = 1.5 kHz)
Difference amplifier (US Mic)	Burr Brown INA105	Nonlinearities	max. 0.001%
		Gain Error	max. 0.01%
QTF	Ralton R38-32.768-12.5-EXT-5PPM	Resonance Frequency	$f_{R}=32,768\;\mathrm{Hz}$
Operational amplifier (QTF)	Texas Instruments 134 PA	Nonlinearities	Typ. 0.00008%
		Bandwidth	8 MHz
Focus Lens (CaF_2_)	Thorlabs LA5817-E	Diameter	1″
		Focal length	100 mm
Lock-In-Amplifier (LIA)	Signal Recovery 7265	Time constant	2 s
		Voltage sensitivity	50 µV

## Data Availability

The data presented in this study are available on request from the corresponding author (accurately indicate status).
